# Knowledge and use of biosimilars in oncology: a survey by the European Society for Medical Oncology

**DOI:** 10.1136/esmoopen-2018-000460

**Published:** 2019-03-06

**Authors:** Rosa Giuliani, Josep Tabernero, Fatima Cardoso, Keith Hanson McGregor, Malvika Vyas, Elisabeth G E de Vries

**Affiliations:** 1 Medical Oncology, San Camillo-Forlanini Hospital, Rome, Italy; 2 Vall d’Hebron University Hospital and Institute of Oncology (VHIO), Universitat Autònoma de Barcelona, Barcelona, Spain; 3 Breast Unit, Champalimaud Clinical Center/Champalimaud Foundation, Lisbon, Portugal; 4 Chief Executive Officer, European Society for Medical Oncology (ESMO), Lugano, Switzerland; 5 Head of Public Policy, European Society for Medical Oncology, Lugano, Switzerland; 6 Department of Medical Oncology, University Medical Center Groningen, University of Groningen, Groningen, The Netherlands

**Keywords:** biosimilars, oncology, prescribers, extrapolation, switching

## Abstract

**Background:**

Biosimilars can potentially improve the sustainability of cancer care; however, uptake is sometimes limited by safety concerns and a lack of understanding of the concept of extrapolation. The European Society for Medical Oncology (ESMO) conducted a survey to assess the current level of knowledge, understanding and comfort of use of biosimilars among prescribers specialised in oncology.

**Methods:**

A 19-question survey was developed using the SurveyMonkey online platform (https://www.surveymonkey.com/). Data collection occurred between September and October 2017 and included paper and online responses.

**Results:**

Overall, 393 responses were received from prescribers. Overall, 49.0% of prescribers use biosimilars in clinical practice and most (79.2%) rate their general knowledge of biosimilars as average to very high. Potential increased risk of immunogenicity remains a significant concern of switching. Gaps in knowledge identified by the survey include biosimilar development, clinical trial design and endpoint selection, and requirements for extrapolation, which should form the focus of future educational initiatives. A substantial demand remains for further educational activities with equal preference for online and face-to-face initiatives. A higher rate of biosimilar use (56.3% vs 46.5%), knowledge of biosimilar development and trial design, and comfort with extrapolation, but a lower knowledge of European Medicines Agency definitions, was found among prescribers from Asia-Pacific versus those from Europe.

**Conclusion:**

Encouraging levels of prescriber use and general knowledge of biosimilars were found, but a substantial need for further education remains. Efforts should be made worldwide to align terms, definitions and guidelines for the development and approval of biosimilars.

**Key questions**

**What is already known about this subject?**

- Biosimilars may potentially reduce care costs and improve patient access.
- However, uptake of biosimilars in oncology has been limited by safety concerns.
- In particular, surrounding switching from a biosimilar to its reference medicine or vice versa, and a lack of understanding around the requirements for development, including extrapolation of indications.

**What does this study add?**

- This European Society for Medical Oncology survey found encouraging levels of prescriber use and general knowledge of biosimilars; however, a substantial need for further education remains, especially for improving prescriber understanding of extrapolation of indications.
- Discrepancies in responses were found among Asia-Pacific and European prescribers.
- Asia-Pacific prescribers appear more confident in their understanding of the biosimilar development process, the concept of extrapolation of indications and switching, but less confident on European Medicines Agency definitions.
- A worldwide effort should be undertaken to align definitions and regulatory standards.

**How might this impact on clinical practice?**

- Educational initiatives focused on the knowledge gaps identified in this survey are essential for successful integration and uptake of biosimilars in oncology, which can potentially improve the sustainability of cancer care by increasing the accessibility of therapeutic and supportive care and providing lower-cost alternatives to their reference medicines.

## Introduction

Cancer treatment has been advanced by, but become reliant on, biologics.^1 2^ Biologics are typically large proteins such as monoclonal antibodies, interferons and recombinant hormones.^3^ Processes for biologic production involve living systems and complex procedures requiring the utmost precision to guarantee final product consistency and quality.^3 4^ These complex manufacturing processes, as well as their long development times, result in biologics being expensive,^2 5^ adding to the already high costs of cancer treatment.

Cancer care cost is rapidly becoming a significant issue driven by rising cancer incidence, ageing populations and the increasing price of treatments.^5^ Patent expiration has occurred or is approaching for many biologics used in oncology.^6^ It has been proposed that the sustainability of cancer care worldwide can potentially be improved through the use of safe and effective biosimilars, which expand the treatment options available to clinicians and patients, increase accessibility of therapeutic and supportive care, and provide lower-cost alternatives to their reference medicines.^2 5 7 8^

A biosimilar is a biologic that matches its reference medicine in terms of quality, activity, safety and efficacy.^9–11^ In 2006, a recombinant human growth hormone (Omnitrope) was the first biosimilar to receive European Medicines Agency (EMA) approval,^12^ followed by EMA approval of biosimilars for epoetin alfa in 2007 and recombinant human granulocyte colony-stimulating factor (rhG-CSF) in 2009.^13 14^ The biosimilar rhG-CSF (Zarxio) was the first biosimilar to receive approval from the US Food and Drug Administration (FDA).^15^

Regulatory requirements for biosimilars are evolving and becoming more familiar among healthcare professionals (HCPs). The EMA, FDA and WHO require substantial evidence demonstrating that a biosimilar matches its reference medicine.^9–11^ The objective of a biosimilar development programme is to demonstrate no clinically meaningful differences based on the ‘totality of evidence’ approach, that is, a comprehensive comparison of the proposed biosimilar and the reference medicine with respect to structure, function, pharmacokinetics, pharmacodynamics, clinical immunogenicity, safety and efficacy.^9–11^ Unlike the standard requirements for drug approval, the development process for biosimilars demands a relatively larger amount of preclinical than clinical data.

Despite the potential for reducing care costs and improving patient access, uptake of biosimilars in oncology has been limited potentially by a lack of understanding of their development and of the regulatory assessment, including requirements for extrapolation of indications.^7^ Extrapolation is the approval, by a regulatory agency, of a biosimilar in one or more indications of the reference biologic without the requirement to carry out clinical trials of the biosimilar in all those indications.^16 17^ If similarity between the biosimilar and its reference biologic is credibly shown through ‘totality of evidence’ in one indication, extrapolation permits approval of the biosimilar in all other indications held by the reference biologic.^18^ Extrapolation has the potential to reduce the costs associated with biosimilar development, increasing access to biologic therapies and reducing cancer care costs.^7^

Uncertainties exist among HCPs regarding switching, the decision to administer a biosimilar in a patient previously treated with the reference biologic or vice versa, and the potential for reduced efficacy or increased immunogenicity in both the oncology and non-oncology settings.^3 19–22^

In order to assess the current knowledge, understanding and comfort of use of biosimilars in oncology, with a particular focus on extrapolation and switching, the European Society for Medical Oncology (ESMO) conducted a survey among its members and attendees at the 2017 ESMO Congress in Madrid, Spain.

## Methods

ESMO developed a 19-question survey using the SurveyMonkey online platform (https://www.surveymonkey.com/), which sought information regarding responders’ use and basic knowledge of biosimilars, understanding of biosimilar development and level of comfort with extrapolation, interchangeability and switching (see online supplementary appendix).

10.1136/esmoopen-2018-000460.supp1 Supplementary data

Data collection occurred between September and October 2017 and included both paper and online responses. During the ESMO 2017 Congress, attendees completed paper copies of the survey; results were then inputted into the SurveyMonkey platform. Additionally, a link to the online survey was sent in an email to ESMO members and their wider professional network. Results were summarised using descriptive statistics.

The survey contained a mixture of checkbox answers and questions asking responders to rank their level of agreement, knowledge, comfort or importance of each statement from 1 to 5 or 10. For these questions, results were pooled and a weighted average (WA) score out of 5 or 10 was assigned. An open comments box at the end of the survey asked responders to provide suggestions for future educational initiatives.

## Results

### Demographics, basic knowledge and use

Overall, 495 responses were collected. Of the 480 responders who mentioned their country, most were from Europe (n=321), then Asia (n=84), America (n=55), Africa (n=13) and Australia (n=7).

These analyses include responses from prescribing physicians only and evaluate responses from all prescribers, European and Asia-Pacific prescribers. Overall, 80.0% (393/491, 4 skipped) of responders were prescribing physicians (Europe: 79.7%, n=255; Asia-Pacific: 87.9%, n=80), with most being ESMO members (92.0%, n=357) and specialised in oncology (94.3%, n=367).

**Figure 1:**
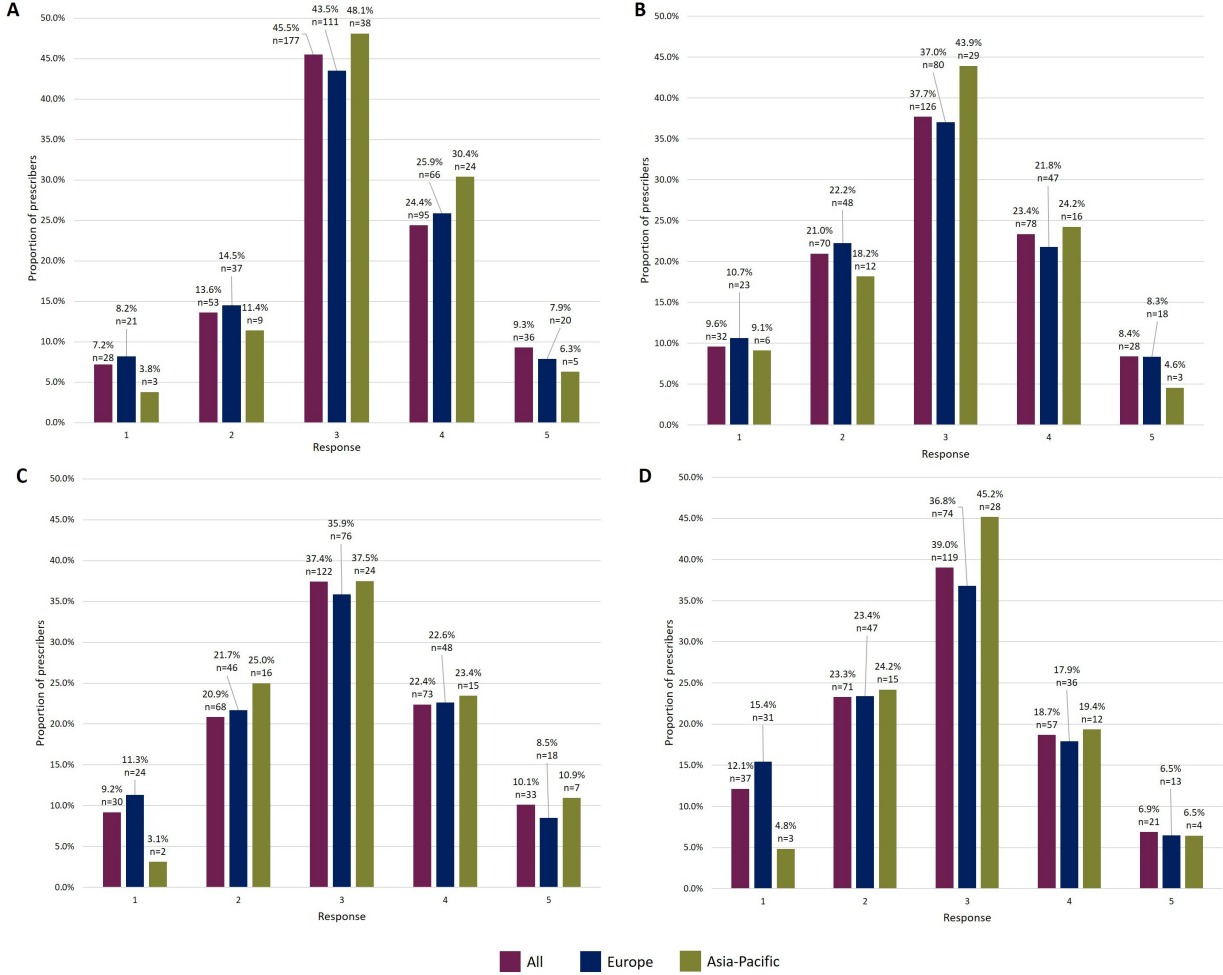
Prescribers’ responses rating their level of knowledge/understanding on a scale of 1–5. Prescribers’ responses, by region, when asked to rate their knowledge/understanding of (A) biosimilars overall; (B) the biosimilar development process and threshold of clinical evidence required for approval; (C) clinical trial design and endpoint selection for biosimilar studies; (D) requirements needed to be met for extrapolation of indications to be granted for a biosimilar.

When asked to rate their overall knowledge of biosimilars, the most commonly selected option on a scale of 1 (very low) to 5 (very high) was option 3 (45.5%, 177/389, 4 skipped; figure 1A). Options 3, 4 and 5 were selected by 79.2% (n=308; sum of responses) of prescribers indicating that most consider themselves to have an average to very high level of knowledge of biosimilars. In total, 74.6% (291/390, 3 skipped) of prescribers were able to identify the most appropriate definition of ‘biosimilar’ (‘highly similar to an approved biological medicine, with no clinically meaningful differences in safety and efficacy profile’). This definition was selected by 77.9% (197/253, 2 skipped) of European and 64.6% (51/79, 1 skipped) of Asia-Pacific prescribers.

**Figure 2:**
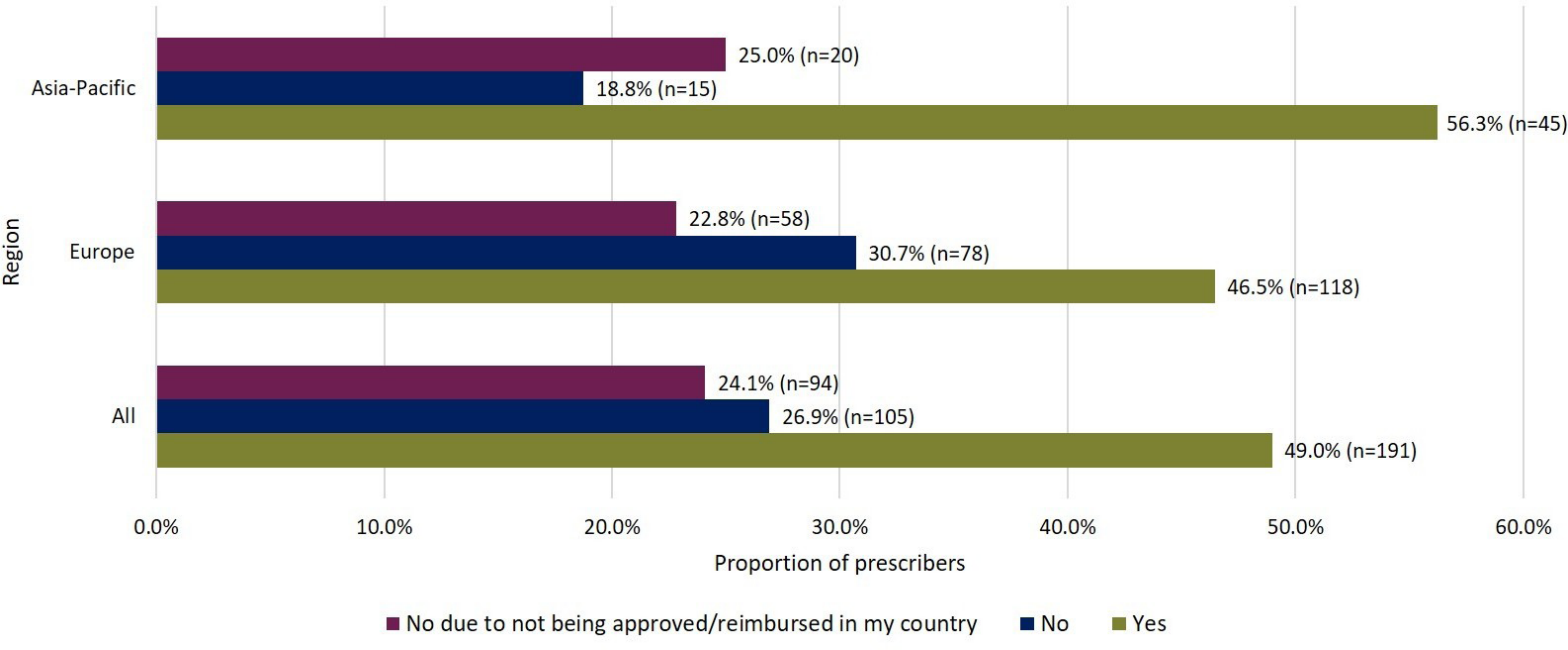
Level of routine use of biosimilars by prescribers in clinical practice to treat patients.

Overall, 49.0% (191/390, 3 skipped) of prescribers use biosimilars in routine clinical practice (excluding clinical trials; figure 2). A higher proportion of prescribers from Asia-Pacific (56.3%, 45/80) use biosimilars versus those from Europe (46.5%, 118/254, 1 skipped; figure 2). Compared with the entire European group, rate of use was lower among prescribers from the UK (31.3%, 10/32). Biosimilars are not used by 24.1% (94/390, 3 skipped) of prescribers due to lack of approval or reimbursement in their country (figure 2).

**Figure 3:**
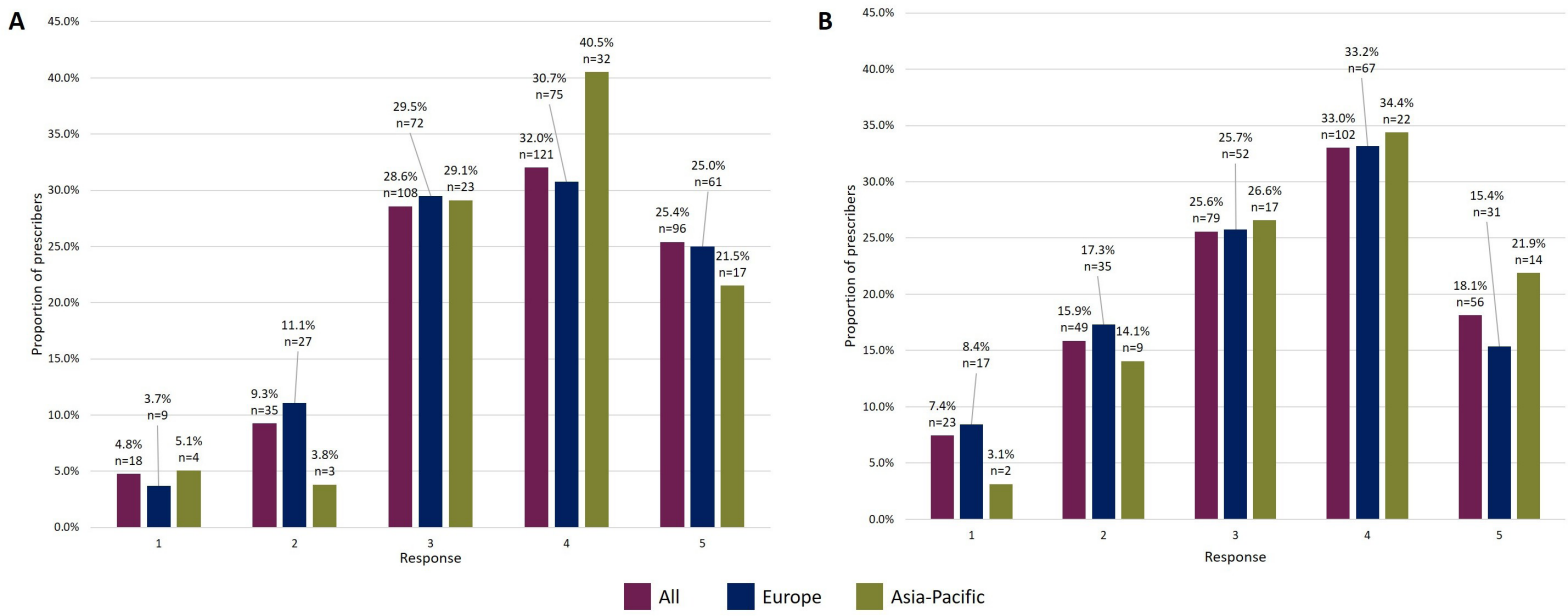
Prescribers’ responses rating their level of comfort on a scale of 1–5.** Prescribers’ responses, by region, when asked to rate their comfort with (A) the concept of using an EMA-approved biosimilar to treat a patient suitable for the reference biologic; (B) using an EMA-approved biosimilar in extrapolated indications that the reference biologic is approved for. EMA, European Medicines Agency.

On a scale of 1 (not at all) to 5 (very), prescribers were asked to rate their comfort with the concept of using an EMA-approved biosimilar to treat a patient suitable for the reference biologic. Options 4 and 5 were chosen by 57.4% (217/378, 15 skipped, sum of responses) of prescribers (figure 3A).

### Biosimilar development

Most prescribers feel they have an average to moderate level of knowledge of the biosimilar development process and the threshold of clinical evidence required for approval of a biosimilar, with 61.1% (204/334, 59 skipped, sum of responses) selecting options 3 and 4 on a scale of 1 (very low) to 5 (very high; figure 1B). Option 3 was the most frequent choice by Asia-Pacific and European prescribers (43.9% vs 37.0%; figure 1B) with the second most commonly selected choices being option 4 (24.2%) and option 2 (22.2%), respectively (figure 1B).

Clinical safety and efficacy data are the best understood data types by all prescribers (WA [out of 5] 3.67), followed by immunogenicity data (WA 3.10). However, the two regions differed on the least understood type of data (Europe: physicochemical, WA 2.64; Asia-Pacific: in vitro, WA 2.36). In addition, clinical study safety (WA [out of 10] 8.80) and efficacy (WA 8.65; table 1) data are considered the most important among prescribers in determining the suitability of a biosimilar for use. The type of data considered least important is physicochemical data demonstrating structural similarity (WA 7.23; table 1).

**Table 1:** Prescribers’ responses rating the importance and sensitivity of different data types in determining the suitability of a biosimilar for use

Type of data (weighted average)	Importance			Sensitivity		
	All	Europe	Asia-Pacific	All	Europe	Asia-Pacific
Physicochemical data demonstrating structural similarity	7.23	7.05	7.30	7.24	7.07	7.56
In vitro and in vivo data demonstrating similarity in biological activity	7.76	7.74	7.66	7.58	7.46	7.82
PK and PD data demonstrating similarity	7.94	7.85	8.10	7.83	7.75	8.10
Clinical study data demonstrating similar efficacy	8.65	8.56	8.72	8.61	8.57	8.75
Clinical study data demonstrating similar safety	8.80	8.78	8.83	8.75	8.72	8.79
Clinical study data demonstrating similar immunogenicity	8.24	8.24	8.10	8.30	8.23	8.41
Clinical study data demonstrating the ability to switch from reference to biosimilar and vice versa without impairing safety or efficacy	8.07	8.02	8.27	8.11	8.02	8.36

Weighted average of prescribers’ responses, by region, on a scale of 1 (not important/sensitive) to 10 (very important/sensitive).

PD, pharmacodynamic;PK, pharmacokinetic.

From three options, 45.2% (146/323, 70 skipped) of prescribers were able to select the most appropriate definition of ‘sensitive indication’ in terms of biosimilar development (‘the population that is most representative of the patients to whom the biologic is most frequently prescribed’). This definition was selected by 42.1% (88/209, 46 skipped) of European and 60.0% (39/65, 15 skipped) of Asia-Pacific prescribers. The second preferred definition (‘a population where product-related differences in clinical performance can be best detected’) was chosen by 31.3% (101/323, 70 skipped) of all prescribers (Europe: 33.5%, 70/209, 46 skipped; Asia-Pacific: 26.2%, 17/65, 15 skipped).

On a scale of 1 (very low) to 5 (very high), responses suggest that most prescribers feel they have an average to moderate level of knowledge regarding clinical trial design and endpoint selection for biosimilar studies, with 59.8% (195/326, 16 skipped, sum of responses) selecting options 3 and 4 (figure 1C). Nearly half of the prescribers (49.7%, 161/324, 69 skipped) chose ‘the endpoint considered most sensitive for detecting differences between the biosimilar and reference biologic, and least influenced by patient or disease-related factors’ as the most appropriate for studies comparing the clinical efficacy of a biosimilar with its reference medicine.

Moreover, 33.4% (108/323, 70 skipped) of prescribers feel that the most appropriate indication for a study comparing the clinical efficacy and safety of a biosimilar with a reference biologic is ‘the indication representing the most sensitive population for detecting any potential difference between the products’. The next most frequent response, selected by 27.9% (90/323, 70 skipped), was ‘comparative efficacy and safety should be studied in every indication of the reference biologic’, suggesting that there is a lack of understanding surrounding the concept of extrapolation of indications.

### Extrapolation of indications

Most prescribers (61.7%, 192/311, 82 skipped) were able to identify the most appropriate definition of ‘extrapolation of indications’ (‘authorisation of a biosimilar in indications of the reference biologic in the absence of specific clinical trial/data for the biosimilar in those indications’). Fewer Asia-Pacific prescribers selected this definition versus European prescribers (53.2% vs 65.4%), although it was the preferred option in both groups.

Despite the high proportion of prescribers being able to define ‘extrapolation of indications’, most consider their understanding of extrapolation to be below average, with 62.3% (190/305, 88 skipped, sum of responses) selecting options 2 and 3 on a scale of 1 (very low) to 5 (very high; figure 1D).

Overall, responses indicate that prescribers feel comfortable using a biosimilar in an extrapolated indication. On a scale of 1 (not at all) to 5 (very), options 3, 4 and 5 were chosen by 76.7% (237/309, 84 skipped, sum of responses) of prescribers (figure 3B). These three options were chosen by 74.3% (150/202, 53 skipped, sum of responses) of European and 82.8% (53/64, 16 skipped, sum of responses) of Asia-Pacific prescribers (figure 3B), indicating that Asia-Pacific prescribers may feel more comfortable with the concept of extrapolation than those from Europe.

### Interchangeability and switching

Respondents were presented with correct EMA definitions of interchangeability and switching, and an incorrect definition of substitution. Only 36.3% (110/303, 90 skipped) of prescribers were able to identify the incorrect definition and the other two correct definitions were also widely chosen (interchangeability: 29.7%, 90/303, 90 skipped; switching: 34.0%, 103/303, 90 skipped). Similar proportions of European (36.0%, 71/197, 58 skipped) and Asia-Pacific (35.5%, 22/62, 18 skipped) prescribers were able to successfully identify the incorrect definition of substitution. However, the same proportion of Asia-Pacific prescribers (35.5%, 22/62, 18 skipped) believed the definition of switching was incorrect.

Regarding switching a patient from a biosimilar to a reference biologic or vice versa, the statement most agreed with by prescribers was “I do not anticipate that switching will have a significant effect on the treatment benefit the patient receives from the product” (WA [out of 5] 3.50). This statement was the most commonly chosen option by both European (WA 3.47) and Asia-Pacific prescribers (WA 3.55). However, among prescribers from Asia-Pacific, there was a similar level of agreement (WA 3.54) with the statement “I do not anticipate that switching will lead to emergence of additional adverse events”.

Among all prescribers, equal levels of concern were expressed for the potential for adverse events (AEs) and increased risk of immune reactions when switching (WA 3.35; table 2). European prescribers were mainly concerned with the potential increased risk of immune reactions (WA 3.39) whereas Asia-Pacific prescribers were primarily concerned with potential AEs (WA 3.35; table 2), despite a high level of agreement with not anticipating additional AEs on a switch in the previous question.

**Table 2:** Prescribers’ responses rating their concern of potential consequences when switching a patient’s treatment from a reference biologic to a biosimilar or vice versa

Potential consequence (weighted average)	All	Europe	Asia-Pacific
Potential loss of clinical efficacy	3.29	3.23	3.30
Potential for adverse events	3.35	3.32	3.35
Potential for increased risk of immune reactions	3.35	3.39	3.17

Weighted average of prescribers’ responses, by region, on a scale of 1 (not at all) to 5 (very).

**Table 2:** Prescribers’ responses rating their concern of potential consequences when switching a patient’s treatment from a reference biologic to a biosimilar or vice versa

Potential consequence (weighted average)	All	Europe	Asia-Pacific
Potential loss of clinical efficacy	3.29	3.23	3.30
Potential for adverse events	3.35	3.32	3.35
Potential for increased risk of immune reactions	3.35	3.39	3.17

Weighted average of prescribers’ responses, by region, on a scale of 1 (not at all) to 5 (very).

### Further education

Overall, 86.7% (195/225, 168 skipped) of prescribers would like ESMO to provide more educational activities concerning biosimilars; a much higher proportion of Asia-Pacific prescribers expressed this (97.9%, 47/48, 32 skipped) versus European prescribers (82.9%, 121/146, 109 skipped).

Prescribers suggested numerous topics for future educational activities, including clinical trial design and endpoints, bioequivalence criteria and studies, approval procedures, principles of pricing and reimbursement, and treatment outcome comparisons between biosimilars and their reference medicines. Communication channels suggested included online educational activities and materials (courses, quizzes, articles, guideline handbooks and updates on key developments; n=56) and face-to-face educational activities (congress sessions, preceptorships, workshops and seminars; n=51). European prescribers communicated a higher interest in receiving training on the efficacy and safety of biosimilars while Asia-Pacific prescribers were more interested in training tailored for developing countries.

## Discussion

This ESMO survey shows that nearly half of prescribers (49.0%) use biosimilars in their clinical oncology practice; lack of approval and reimbursement is a barrier to use. Responses suggest that most prescribers (79.2%) feel they have an average to very high level of general biosimilar knowledge, with nearly three quarters (74.6%) able to identify the most appropriate definition of ‘biosimilar’. Overall, 57.4% of prescribers feel comfortable using an EMA-approved biosimilar.

Most prescribers feel they only have an average to moderate level of knowledge about biosimilar development, the level of clinical evidence required for a biosimilar approval, clinical trial design and selection of endpoints; these therefore present as topics for future educational activities. Indeed, less than half (45.2%) of prescribers were able to identify the most appropriate definition of ‘sensitive indication’. Uncertainties were also demonstrated in differentiating ‘interchangeability’, ‘substitution’ and ‘switching’. Despite nearly two-thirds of prescribers being able to identify the most appropriate definition of ‘extrapolation of indications’, most rated their understanding of the requirements for extrapolation of indications as below average. However, most prescribers feel comfortable using a biosimilar in an extrapolated indication. Therefore, it seems that many prescribers trust and accept the scientific principle of extrapolation though may not fully understand it.

The main concerns oncology prescribers have with switching are the potential for AEs and increased risk of immune reactions. Currently, the majority of data available on switching are from real-world and clinical studies in immune-mediated inflammatory diseases, such as rheumatoid arthritis, inflammatory bowel disease and psoriasis, which have found no clinically meaningful effects when switching between a biosimilar and its reference biologic.^23–42^ Presently, there is one published study on switching in oncology, which reported no meaningful differences in efficacy, safety or immunogenicity when switching biosimilar rhG-CSF with its reference biologic to prevent severe neutropenia in patients with breast cancer undergoing myelosuppressive chemotherapy.^43^ A recent systematic literature review of 90 switching studies, treating 14 disease indications and enrolling over 14 000 patients and healthy volunteers, concluded that there is little risk of increased immunogenicity or treatment-related AEs, or reduction in efficacy, when switching between reference medicines and biosimilars.^21^ An increase in the confidence of switching biosimilar medicines with their reference biologics among the oncology community, like in other disease areas, may occur after increased availability of results from further real-world data and world-evidence studies that will ultimately help to guide clinical decision-making.

Regarding knowledge, use and comfort with biosimilars, some differences in responses were found between European and Asia-Pacific prescribers. First, a higher rate of biosimilar use in routine clinical practice was found among Asia-Pacific (56.3%) versus European prescribers (46.46%). This may be a result of numerous regulatory bodies in Asia heavily investing in accelerated development of biosimilars.^44^ Furthermore, a lower rate of biosimilar use was found among prescribers from the UK (31.3%) compared with the whole group of European prescribers. Responses also suggest that Asia-Pacific prescribers may be more confident in their understanding of biosimilar development than European prescribers.

However, in most questions concerning EMA definitions, a lower proportion of Asia-Pacific prescribers answered correctly versus European prescribers. The only instance where this was not the case was the definition of ‘sensitive indication’, correctly answered by 60.0% of Asia-Pacific and 42.1% of European prescribers, indicating that Asia-Pacific prescribers may have a better understanding of the clinical evidence required for a biosimilar to gain approval and clinical trial design. Lastly, responses suggest that prescribers from Asia-Pacific are more comfortable with the concept of extrapolation than those from Europe.

These differences in responses, particularly regarding EMA definitions, may be a result of differing guidelines available on biosimilars in each region. Europe follows standardised regulations outlined by the EMA for biosimilar approval whereas the regulations in Asia-Pacific countries differ greatly in the data types required.^44^ Since 2008, guidance ensuring the quality, safety and efficacy of biosimilar medicines has been available in the Asia-Pacific region.^45^ EMA guidance was followed initially in many countries in the Asia-Pacific region prior to them implementing their own individual guidelines.^45^ Countries including South Korea, Japan and Malaysia established their own guidance by taking elements from EMA and WHO guidelines.^45–48^ In August 2016, India updated its initial guidance originally published in 2012 to ensure a clearer and more thorough regulatory pathway, and to make parallel with other guidelines available around the world.^49^

Currently, the requirements outlined by regulatory authorities in different regions regarding biosimilar approval vary.^9 47^ A worldwide effort should be undertaken to align definitions and standards for the development and approval of biosimilars. This could potentially reduce confusion surrounding scientific terms and concepts, lead to better understanding of the biosimilar development process and ultimately increase accessibility and affordability of cancer care.^8^

Differences in responses were also noted between prescribers specialised in oncology and haematology. Of the 393 prescribers who responded, 22 (5.6%) were specialised in haematology. Overall, knowledge of biosimilar development and trial design, as well as understanding and comfort of extrapolation of indications, were similar between prescribers specialised in oncology and haematology. However, a higher proportion of haematology versus oncology prescribers feel they have a high to very high level of comfort using an EMA-approved biosimilar (72.8% vs 57.4%) and use biosimilars in routine practice (63.6% vs 49.0%).

Overall, the level of prescriber knowledge on biosimilars ascertained by this survey is encouraging. However, a substantial need for continued education emerged as well. Future efforts should focus in particular on improving prescriber understanding of extrapolation of indications as well as physicochemical data, which was found to be the least understood data type in determining the suitability of a biosimilar for use when in fact it is considered by regulatory authorities as the most determinant data type required. This survey found a substantial demand among prescribers for educational activities and materials regarding biosimilars, especially in Asia-Pacific. Responses suggest that preference is fairly even between online (56 responses) and face-to-face (51 responses) educational activities. The low response to this question is potentially due to its open comment box design which is more demanding for respondents and, being the final question, it is feasible that responders ran out of time. Prescribers from the two different regions had differing preferences for topics that future training initiatives should focus on. European prescribers displayed a high interest in receiving training on the efficacy and safety of biosimilars, while many from Asia-Pacific conveyed an interest for more training adapted for developing countries. ESMO is undertaking a range of educational initiatives including two previous sessions during the ESMO 2017 meeting in Madrid and ESMO Asia 2017, and another two Colloquia during the 2018 annual meeting in Munich, Germany, and ESMO Asia 2018, to improve the understanding of biosimilars within the community. ESMO is also working on developing patient materials to help their understanding of biosimilars.

Limitations of the survey include the fact that no hypothesis was tested and the questionnaire was purely developed to document the current level of biosimilar knowledge, use and comfort; not all replies were complete, so data could not be analysed in its entirety; and responses were limited to ESMO members, their wider network and participants at the ESMO 2017 Congress, so may not accurately represent all prescribers worldwide.

## Conclusion

In conclusion, this survey conducted by ESMO found an encouraging level of prescriber use and general knowledge of biosimilars in oncology; however, need for further education remains. Future educational initiatives should focus on improving prescriber understanding of extrapolation of indications as well as physicochemical data.

Lastly, some differences in responses between European and Asia-Pacific prescribers may be attributed to differences in guidance available in the two regions. Efforts should be made worldwide to align definitions and regulatory standards for the development and approval of biosimilars. Continued education will lead to more informed discussion and decision-making regarding biosimilars, which will help their successful integration and uptake in oncology.^8^
