# Supplementary material for: Knowledge and use of biosimilars in oncology: a survey by the European Society for Medical Oncology
Source: ESMO Open. 2019 Mar 6;4(2):e000460. doi: 10.1136/esmoopen-2018-000460 (PMC6435239; doi:10.1136/esmoopen-2018-000460)
Supplement: Supplementary data [file esmoopen-2018-000460supp001.pdf]

ESMO Survey on Biosimilars in Oncology

We would very much appreciate if you took 10 minutes of your time to complete the following survey.

Background

Are you a prescribing physician: ☐ Yes ☐ No

ESMO member: ☐ Yes ☐ No

Country of practice: ______________

Q1. What is your area of specialty? ☐ Oncology ☐ Hematology

Q2. Overall, how would you rate your knowledge of biosimilars? (1 = very low; 5 = very high)? ______________

Q3. Which of the following most accurately describes a biosimilar?

- ☐ A biological medicine that is identical to an approved biological medicine, with identical safety and efficacy
- ☐ A biological medicine that is highly similar to an approved biological medicine, with no clinically meaningful differences in safety and efficacy profile
- ☐ A biological medicine that is similar to an approved biological medicine, but with an improved safety and efficacy profile
- ☐ A biological medicine that is similar to an approved biological medicine, but with more uncertain safety and efficacy profile

Q4. Do you routinely use a biosimilar in your clinical practice to treat patients (excluding in clinical trials)?

☐ Yes ☐ No ☐ No, because biosimilars are not approved/reimbursed yet in my country

Q5. In principle, how comfortable are you with the concept of using a biosimilar, approved by EMA, to treat a patient suitable for the reference biologic? (1 = not at all comfortable; 5 = very comfortable) ____________

Biosimilar development and evidence

Q6. How would you rate your knowledge of the biosimilars development process and threshold of clinical evidence required for approval? (1 = very low; 5 = very high) ________________

Q7. How would you rate your understanding of the following kinds of data that are commonly generated for biosimilars? (1 = very low; 5 = very high)

- ☐ Physicochemical data (e.g. molecular structure analyses, glycosylation data, etc.)
- ☐ *In vitro* data (e.g. cell-based functional assays, receptor binding)
- ☐ *In vivo* data (e.g. biological activity in animal models)
- ☐ Pharmacokinetic data (e.g. C_{max}, AUC, etc.)
- ☐ Pharmacodynamic data (e.g. effect on pharmacodynamic markers)
- ☐ Clinical efficacy and safety data
- ☐ Immunogenicity data

Q8. Please rate the following in terms of how important you feel they are in determining the suitability of a biosimilar for use? (1 = not important; 10 = very important)

- ☐ Physicochemical data demonstrating structural similarity
- ☐ *In vitro* and *in vivo* data demonstrating similarity in biological activity
- ☐ Pharmacokinetic and pharmacodynamic data demonstrating similarity
- ☐ Clinical study data demonstrating similar efficacy
- ☐ Clinical study data demonstrating similar safety
- ☐ Clinical study data demonstrating similar immunogenicity
- ☐ Clinical study data demonstrating the ability to switch from the reference biologic to the biosimilar and vice versa to the without impairing safety or efficacy

Q9. What does the phrase 'sensitive indication' mean in terms of biosimilar development?

- ☐ A population where product-related differences in clinical performance can be best detected
- ☐ The population that is most representative of the patients to whom the biologic is most frequently prescribed
- ☐ The population in which the most robust dataset exists for the reference biologic

Q10. Which of the following endpoints do you think is most appropriate to use for studies of the comparative clinical efficacy of a biosimilar with a reference biologic?

- ☐ The primary endpoint that was used in the phase III trial with the reference biologic
- ☐ The endpoint considered most sensitive for detecting differences between the biosimilar and reference biologic, and least influenced by patient- or disease-related factors
- ☐ The endpoint most strongly reflective of the clinical benefit of the biologic (e.g. overall survival or progression-free survival rates)

Q11. Please rate the following in terms of how sensitive you think they are in determining the suitability of a biosimilar for use? (1 = not important; 10 = very important)

- ☐ Physicochemical data demonstrating structural similarity
- ☐ *In vitro* and *in vivo* data demonstrating similarity in biological activity
- ☐ Pharmacokinetic and pharmacodynamic data demonstrating similarity
- ☐ Clinical study data demonstrating similar efficacy
- ☐ Clinical study data demonstrating similar safety
- ☐ Clinical study data demonstrating similar immunogenicity
- ☐ Clinical study data demonstrating the ability to switch from the reference biologic to the biosimilar and vice versa to the without impairing safety or efficacy

Q12. How would you rate your knowledge of the clinical trial design (rationale, population, etc) and selection of endpoints for studies of biosimilars? (1 = very low; 5 = very high) ___________

Q13. Which of the following indications do you think is most appropriate to select for studies of the comparative clinical efficacy/safety of a biosimilar with a reference biologic that has multiple indications?

- ☐ The indication relating to the largest phase III trial with the reference biologic
- ☐ The indication for which the reference biologic is most frequently prescribed
- ☐ The indication representing the most sensitive population for detecting any potential difference between the products
- ☐ The first indication which the reference biologic was approved
- ☐ Comparative efficacy and safety should be studied in every indication of the reference biologic

Extrapolation of indications

Q14. Which of the following accurately describes the concept of 'extrapolation of indications' for biosimilars?

- ☐ Authorization of a biosimilar in indications of the reference biologic in the absence of specific clinical trial/data for the biosimilar in those indications
- ☐ Authorization of a biosimilar for use in an indication that is similar to one in which it has already demonstrated clinical comparability

Q15. How would you rate your understanding of the requirements that need to be met for extrapolation of indications to be granted for a biosimilar? (1 = very low; 5 = very high) ___________

Q16. Biosimilar X has shown match with regards to physicochemical (including identical amino acids sequence), preclinical, pharmacokinetic and pharmacodynamic properties to a reference biologic, and similar clinical efficacy and safety in one of the reference biologic's indications. Provided that the biosimilar X was approved by EMA for all indications of the reference biologic, how comfortable would you feel in using biosimilar X in other indications that the reference biologic is approved for? (1= not at all comfortable; 5 = very comfortable) _____________

GOOD SCIENCE
BETTER MEDICINE
BEST PRACTICE

Interchangeability and switching

Q17. Which of the following definitions is incorrect, based on European Medicines Agency definitions?

- ☐ **Interchangeability** = the possibility of exchanging one medicine for another medicine that is expected to have the same clinical effect
- ☐ **Switching** = when the prescriber decides to exchange one medicine for another medicine with the same therapeutic intent
- ☐ **Substitution** = the practice of dispensing one medicine instead of another equivalent and interchangeable medicine at pharmacy level, after consulting the prescriber

Q18. Please indicate how strongly you agree or disagree with the following statements regarding switching a patient from a biosimilar to a reference product or vice versa (1 = strongly disagree; 5 = strongly agree)

- ☐ I **do not** anticipate that switching will have a significant effect on the treatment benefit the patient receives from the product
- ☐ I **do not** anticipate that switching will lead to emergence of additional adverse effects
- ☐ I **do not** anticipate that switching will lead to harmful immunogenicity

Q19. How concerned are you about each of the following when switching a patient's treatment from a reference product to a biosimilar or vice versa? (1 = not at all concerned; 5 = very concerned)

☐ Potential loss of clinical efficacy ☐ Potential for adverse events ☐ Potential for increased risk of immune reactions

Would you like ESMO to provide more educational activities in the biosimilars area?

☐ Yes ☐ No

If you replied Yes above, please specify the type of educational activities that you would like ESMO to organize.

Please feel free to add comments about the topics presented in this survey or any other topics related to biosimilar medicines

Thank you for completing the survey
